# An investigation of mHealth and digital health literacy among new parents during COVID-19

**DOI:** 10.3389/fdgth.2023.1212694

**Published:** 2024-01-25

**Authors:** Lorie Donelle, Brad Hiebert, Jodi Hall

**Affiliations:** ^1^ College of Nursing, University of South Carolina, Columbia, SC, United States; ^2^ Arthur Labatt Family School of Nursing, Western University, London, ON, Canada; ^3^ School of Nursing, Fanshawe College, London, ON, Canada

**Keywords:** maternal health, parenting, mHealth, digital health, digital literacy, pandemic

## Abstract

**Introduction:**

Especially during the COVID-19 pandemic, parents were expected to understand increasingly sophisticated information about health issues and healthcare systems and access online resources as a part of their caregiving role. Yet little is known about parents' online digital technology use and digital health literacy skill. This study aimed to investigate parents' digital technology use, their self-reported digital health literacy skill, and demographic information as potential factors influencing their use of digital technologies.

**Methods:**

An online survey utilizing convenience sampling was administered to new parents during the COVID-19 pandemic that inquired about their demographic information, digital technology use, and digital health literacy skills within Ontario, Canada.

**Results:**

A total of 151 individuals responded to the survey; these were primarily mothers (80%) who self-reported as white (72%), well-educated 86%), heterosexual (86%) females (85%) with incomes over $100,00 per year (48%). Participants reported consistent and persistent online activity related to their parenting role and mostly via mobile smartphone devices (92%). Participants had moderate to high digital health literacy skills, greater than the Canadian national average. Almost half of participants reported negative health and well-being consequences from their digital online behaviours. There were no significant relationships between technology use, digital health literacy skill, and demographic variables.

**Discussion:**

The COVID-19 pandemic has reinforced the need for and importance of effective and equitable digital health services. Important opportunities exist within clinical practice and among parenting groups to proactively address the physical and mental health implications of digital parenting practices. Equally important are opportunities to insert into clinical workflow the inquiry into parents' online information-seeking behaviours, and to include digital health literacy as part of prenatal/postnatal health education initiatives.

## 1 Introduction

Within our data-driven health care system, individuals, patients, and health care providers require proficiency with health information technology and adequate health and digital health literacy skills (1). In support of self and family care, parents are expected to understand increasingly sophisticated information about health issues and health care systems and are accessing online health information, services, and supports as a critical component of their caregiving role (2, 3). The ability to self-care and provide caregiving to others is predicated, in part, on parents' ability to access and appraise reliable and timely information. The challenge for many online information seekers, including parents, is the overwhelming volume of information, the indeterminate quality of said information, and the increasingly sophisticated information about health issues and health care systems (3, 4).

Parents' ability to navigate resources to support their caregiving role is influenced by their health literacy and digital health literacy skills. Health literacy is defined as the degree to which people are able to access, understand, appraise, and communicate information to engage with the demands of different health contexts in order to promote and maintain good health (5). While there appears to be no Canadian national assessment of parents' heath literacy skill, concernedly, 60% of Canadians lack the skills and ability to make appropriate health care decisions (6). Comparatively, a parental health literacy skill survey conducted in the USA showed that almost 30% of parents demonstrated inadequate health literacy skills and were not able to correctly complete health forms or understand over-the-counter medication administration instructions, and were three times more likely to be without health insurance (7). Berkman et al. (2011) conducted a systematic review of health outcomes in relation to health literacy and showed that low health literacy contributes, in part, to greater health service utilization, lower rates of cancer screening, poor vaccine uptake, increased risk of inappropriate medication administration and adherence, and limited ability to interpret labels and health messages (8).

The ability to read, write, listen, speak, calculate, critically analyze, communicate, and interact with health information becomes even more complex within the digital health context. Health care organizations and governmental sectors offer a range of health-related resources (e.g., policies, forms, patient instruction, health education materials) online—some exclusively available within a virtual care setting (9). Our reliance on virtual health and social care services further emphasizes the need for adequate digital health literacy skills to access health and social services. Digital health literacy is defined as the ability to appraise health information from electronic sources and the ability to apply the information to addressing or resolving a health issue (10). Therefore, to fully and equitably participate within digital health spaces, individuals/parents require sufficient skills at the intersection of civic, digital, health, and digital health literacies that reflect the abilities to: read and understand general information (traditional literacy and numeracy), safely and securely use data and digital devices (computer literacy), critically understand and assess media messages (media literacy), discern what is reliable and valid information (scientific literacy), and search for information (information literacy) to have the knowledge necessary to promote their own health and to be able to navigate the health care system (health literacy) (11–13).

To our knowledge, publicly available information specific to parents' digital health literacy skill is not available. However, according to data from a national digital health literacy survey, Canadians (16–64 years) have a range of inadequate to adequate digital health literacy skills but, on average, have sufficient skill; meanwhile, younger Canadians (16–34 years) report as having significantly greater skill than Canadians 35 years and older (14). An inability to access, appraise, and accurately apply online health information within a personal context is associated with delayed access to health services and increases the need for costly health care services (15). During the COVID-19 pandemic, researchers reported over 92% of Canadians aged 15 and older were “online,” which was only slightly more online engagement than in the year 2018, which was at 91% (16). Almost one in four Canadians (24%) had either no engagement or only very limited engagement with the Internet and digital technologies in 2019. Younger (age 15–34 years) Canadians and those with higher educational attainment (bachelor's degree or higher) tended to be proficient (22%) or advanced (34%) Internet users (17).

Internet use during COVID-19 increased among Canadians living in a household with children under 18 years of age (98%) (17). Reported online activity included increased online shopping and communication using instant messaging and social networking apps and voice or video calling. Not surprisingly, online health information seeking increased during the pandemic; approximately 70% of Canadians accessed the Internet for health information and almost 25% used online tools to enhance their health and well-being (e.g., tracking their nutrition and fitness) (17). Public awareness to the challenges of Internet use was also heightened, and awareness of online safety was demonstrated through the use of safety precautions, such as restricting or refusing to disclose geographical location, denying the use of personal data for advertising, or adjusting privacy settings to limit personal information (17).

Parents appear to be prolific users of online resources to access information, to connect with others for support, and to access health-related services (2). In an investigation of digital technology use among mothers in Australia, researchers found that pregnant women and mothers of young children place high value on the information generated from digital sources (18). An important consideration derived from this study suggests it is essential to understand how women use digital technology to obtain information and how women are creators of information accessible to others in their capacity as mothers (19).

Studies of new mothers active on Facebook after giving birth noted a relationship between high social media activity and increased stress levels and feelings of isolation, and reported greater depressive symptoms among mothers seeking external validation about their parenting practices (20–24). Furthermore, a survey of mothers with young children examining the relationship between online resources and infant feeding practices demonstrated that time spent on mothering websites was negatively correlated with the intention to breastfeed their child during the transition to parenting period (25).

The number of pregnancy apps is rapidly increasing, and they are playing an important role in the self-health promotion of women and infants. Lee and Moon (2016) utilized a content evaluation to conduct a descriptive study exploring the use of mobile apps for pregnancy, birth, and childcare. Credibility of the health information was evaluated for 47 apps most frequently used by their study participants. They concluded that the quick provision of health information was desired and seen as a motivator, but oftentimes, credible professional information was sorely lacking (26). Apps have become an important information source for pregnant women, more frequently used when searching for information concerning signs of risk and disease (26, 27). Daly et al. (2019) conducted a systematic review to appraise the quality of health information about decreased fetal movement provided through mobile apps intended for use during pregnancy. Of the assessed apps, 24 mentioned decreased fetal movement, but few apps explicitly linked decreased fetal movement to stillbirth or other specific adverse outcomes, and lacked clear instructions as to what to do based on results (28).

An overarching concern related to access and use of online health resources is the trustworthiness of the information and tools, and the potential impact of these resources on the health and well-being of individuals and families. Before COVID-19, parents, in a qualitative study on their use of technology to support their parenting role, described prolific online information-seeking, especially when an appointment with a health care provider was unavailable and/or if information and services were only available through online resources (2). In favor of information immediacy or in the absence of accessible medical advice (lack of a primary care provider or information needed outside of clinical service hours), evidence indicates that parents will overlook concerns regarding information quality and privacy violations (2).

In essence, many parents are using mobile devices (smartphones) to access the Internet and social media apps for health information, services, and supports. The COVID-19 pandemic has further accelerated the acceptability and access to virtual care services. In acknowledging that digital health literacy skills are foundational to understanding, appraising, and communicating information to make informed health care decisions for self and family, the lack of research assessing digital health literacy skills generally and specifically among new parents is surprising. The aim of this study was to investigate parents' frequency and nature of digital technology use and their self-reported digital health literacy skill. This study considered gender, education, age, and geographic location as potential factors influencing parents' use of digital technologies.

## 2 Methods

Using a non-experimental, cross-sectional design, this study investigated new parents' use of personal digital devices (hardware and software) and examined the relationships of digital health literacy, education, income, and geographic location (urban/rural) among first-time parents within Ontario, Canada. An online survey utilizing a non-probability, convenience sampling strategy (29) was administered to new parents during the COVID-19 pandemic to address the following research questions: (1) What available digital devices and tools are used by new parents? (2) What is the frequency/nature of use to support their parental caregiving role? and (3) What is the self-reported digital health literacy skill among new parents? Our research protocol that included details of our data collection, privacy, and security practices was approved by the university research ethics board (REB) where the authors were employed.

### 2.1 Participant recruitment

Individuals 16 years of age or older with at least one child two years of age or younger were welcome to participate in the study. Participants' implied consent was obtained prior to data collection as indicated by checking “yes” to a question at the end of the online letter of information that confirmed their review of the letter and consent to participate in the study. Participants who checked “yes” were then directed to the online survey, while participants who checked “no” were directed to a thank you page and were not exposed to the online survey. Online advertisements were used to generate awareness across a range of social media platforms, including Twitter and Facebook. Completion of the online survey took approximately 15 min and could be completed at any time or location at the participants' convenience. At the outset of the survey, potential participants were notified about the option to enter into a draw for a $50.00 gift card as part of the informed consent process. With their consent, survey participants were entered into a $50.00 gift card draw at the close of the survey data collection. Three participants from those who had consented to be included were randomly selected in a draw to receive the gift card.

All participants were invited to enter into a $50 gift card draw following the conclusion of the survey.

### 2.2 Data collection

Data collection occurred during the first year of the COVID-19 pandemic from January—November 2020 inclusive. An online survey was administered using the Qualtrics platform to assess participants' demographic profiles, the type and frequency/nature of use of digital devices, and participants' self-reported digital health literacy skill. Surveys included standardized instruments and researcher-developed questions. The main survey questions were derived from standardized research instruments and have been validated in previous studies. A set of questions to help us describe the demographic characteristics of the participants were added to the survey, including age, education, gender, sexual orientation, marital status, income, and place of residence.

#### 2.2.1 Data collection instruments

1. Type and nature of digital technology use items adapted from the Canadian Internet Use Survey 2018 (CIUS) (30). The CIUS survey focused on measuring individuals' use of digital technologies, the Internet, and online behaviours, including those related to social media, e-commerce, online government services, online work, digital skills, streaming content, and security, privacy, and trust as they relate to the Internet. Various self-reported response options were provided to respondents according to the question being asked. For particulars on questions asked and response options consult aforementioned reference (30). The survey also measured household access to the Internet.
2. Digital health literacy skill was measured by the eHealth Literacy Scale (eHEALS) (12). The scale consists of eight items. The items measure the self-reported ability to find, assess, and use online information in order to make informed health-related decisions. Answers were graded on a five-point Likert scale, with Grade 1 denoting “strong disagreement” and Grade 5 denoting “strong agreement.” The total eHEALS score was obtained by summing grades for each item. The total scores ranged from 8 to 40, with higher scores denoting higher reported eHealth literacy. Scores of 8 to 20 reflect inadequate digital health literacy knowledge and skill; individual scores ranging from 21 to 26 are problematic; and scores from 27 to 40 represent sufficient digital health literacy knowledge and skills (31). The original validation study showed the internal consistency of eHEALS of 0.88 as measured by the Cronbach's alpha coefficient (10). Additional studies testing the psychometric properties of the eHEALS confirm that it is a reliable and valid instrument (31–33).

### 2.3 Data analyses

Data was analyzed using SPSS version 28. None of the analytical features of the Qualtrics platform were utilized for our data analysis. Data generated from the survey were subjected to descriptive, relational, and comparative analyses to better understand the distributions of variables of interest and to determine what differences existed between groups within the sample. Descriptive analyses were conducted to describe participants' demographic profiles, nature of care providers responsible for participants' postpartum care, digital health literacy skill (eHEALS scale responses), and the nature of participants' Internet and digital technology use in general and as it directly relates to their role as a parent. Correlational analyses were conducted to determine what relationships existed between participants' eHEALS score, age, and belief that parenting success is related to technological skill. Chi-square analyses were conducted to determine if participants' belief that parenting success is related to technological skill was associated with their education, annual income, employment status, or community size. An independent t-test was conducted to determine the difference in eHEALS scores between participants who did and did not believe they needed to be skilled with digital technology to be successful at parenting. One-way analyses of variance (ANOVA) using Tukey's HSD post-hoc test were conducted to determine if there was any difference in eHEALS scores based on participants' education, household income, or community size. An alpha of.05 was used for all correlational and comparative analyses to identify statistically significant results.

## 3 Results

### 3.1 Demographic characteristics of participants

A total of 151 participants responded to the online questionnaire, of whom 121 (80%) self-identified as mothers and 14 (9%) as fathers; 16 (11%) elected not to respond. Participants also self-identified as female (*N* = 128, 85%), male (*N* = 14, 9%), and gender fluid (*N* = 1, 1%), and 8 (5%) preferred not to respond. The mean age of participants was 32.57 years (*s* = 4.36). Most participants identified as heterosexual (*N* = 130, 86%) while 14 (9%) identified as LGBT+, and 95% (128) were married or living common-law. The majority of participants were white (*N* = 108, 72%), and 131 (86%) had attained college-, university-, or graduate-level education. Just over half of participants were employed full-time (*N* = 92, 61%), 21 (14%) participants claimed part-time or casual employment, 18 (12%) participants were on leave from their workplace, and 5 (3%) were unemployed. Annual household income for 13 (9%) participants was below $50,000, and almost half (*N* = 72, 48%) of participants reported greater than $100,000/year income. About half (*N* = 71, 47%) of participants lived in communities larger than 100,000 people and about 1 in 4 participants (*N* = 35, 23%) lived in communities of less than 10,000 people (see Table 1).

**Table 1:** Demographic characteristics of participants (*N* = 151).

	Mean	*s*
Participant age	32.57	4.36
	Count (*N*=)	%
Parent role		
Mother	121	80%
Father	14	9%
Prefer not to answer	16	11%
Gender identity		
Female	128	85%
Male	14	9%
Gender fluid	1	1%
Prefer not to answer	8	5%
Sexual orientation		
Heterosexual	130	86%
LGBT+	14	9%
Prefer not to answer	7	5%
Racial and ethnic identity		
White	108	72%
Multiracial	2	1%
Indigenous	1	1%
East Indian	1	1%
Korean	1	1%
Latin	1	1%
South Asian	1	1%
Chinese	1	1%
Preferred not to answer	35	23%
Highest level of education		
High school or lower	4	3%
Community college/apprenticeship	33	22%
University undergraduate degree	46	30%
University graduate degree	52	34%
Prefer not to answer	16	11%
Employment status		
Employed full-time	92	61%
On leave	18	12%
Employed part-time	17	11%
Unemployed	5	3%
Casual	4	3%
Prefer not to answer	15	10%
Marital status		
Married/common-law	128	95%
Single, never married	4	3%
Separated	2	1%
Prefer not to answer	17	11%
Annual household income		
$49,999 or lower	13	9%
$50,000 to $99,999	48	32%
$100,000 or higher	72	48%
Do not know	2	1%
Prefer not to answer	16	11%
Population of city/town/village		
Less than 10,000 people	35	23%
10,000 to 29,999 people	12	8%
30,000 to 99,999 people	17	11%
100,000 people or more	71	47%
Prefer not to answer	16	11%
Weeks of gestation at most recent delivery		
35 weeks or less	13	9%
36 to 39 weeks	57	38%
40 + weeks	60	40%
Prefer not to answer	21	14%

Individuals' clinical postpartum care was provided by family practitioners (*N* = 61, 51%), obstetric specialists (*N* = 56, 47%), and midwifes (*N* = 49, 41%); support was also provided by doulas (11%), and most individuals reported giving birth between 36 and 40 weeks gestation (*N* = 117, 78%).

### 3.2 Participants' digital health literacy skill

Digital health literacy reflects individuals' self-reported ability to find, assess, and use online information to make informed health-related decisions for self and others. Participants reported, on average, moderate to high digital health literacy skill, with a mean eHEALS scale total of 32.02 (*s* = 4.35). Most participants reported having sufficient (*N* = 100, 90.1%) digital health literacy knowledge and skill, with the remainder reporting problematic (*N* = 9, 8.1%) or inadequate (*N* = 2, 1.8%) digital health skill. Cronbach alpha scores (0.88) were acceptable. There was no statistically significant relationship between participants' digital health literacy skill and age (*r* = .08, *N* = 109, *p* = .41). Analyses of variance indicated that there was no significant effect on participants' eHEALS scale total based on their education [*F*(4, 104) = 1.49, *p* = .21], household income [*F*(4, 104) = .89, *p* = .48], or community size [*F*(3, 105) = .33, *p* = .81].

**Figure 1:**
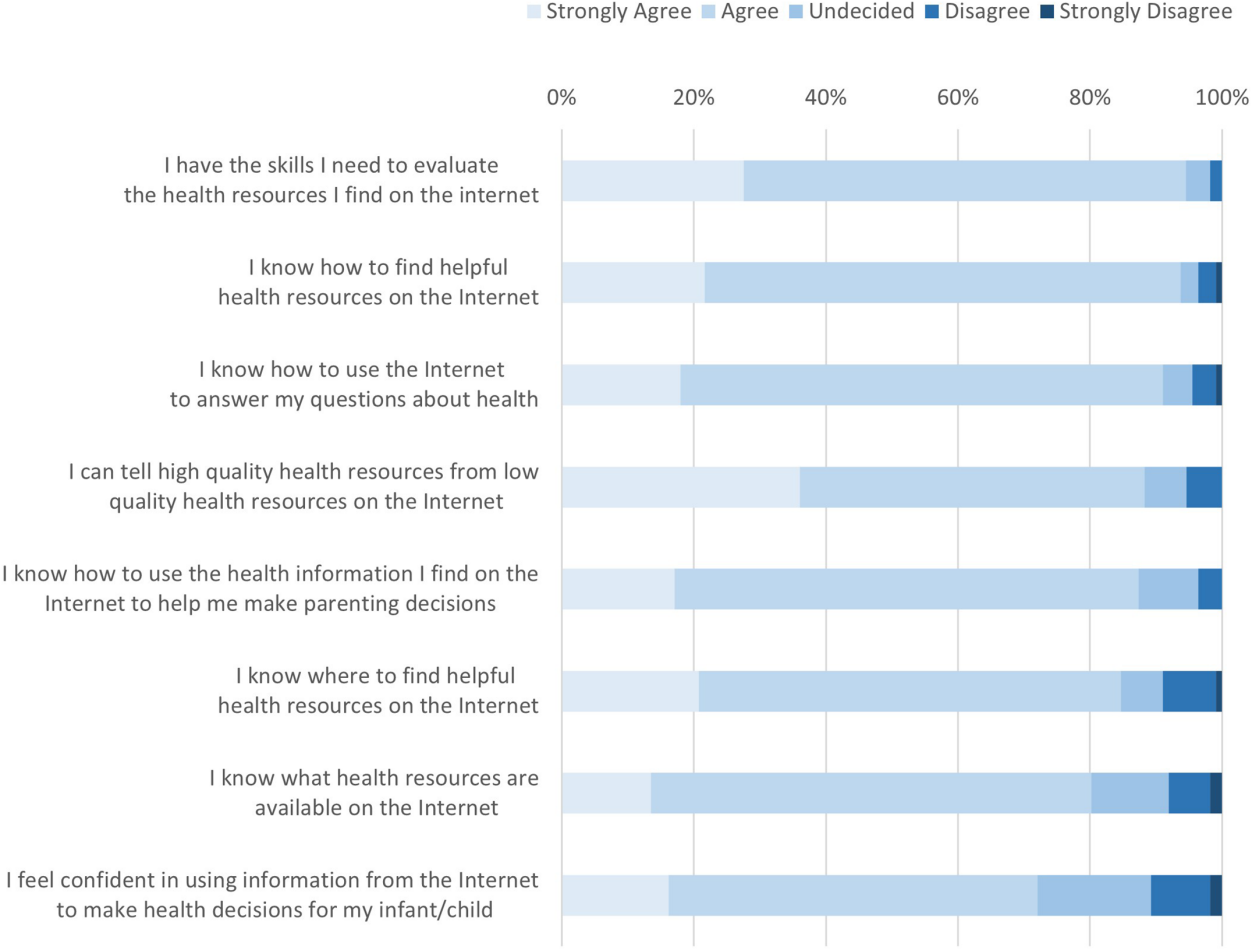
eHEALS scale responses, by scale item (*N* = 109).

Notable is that almost 85% of participants agreed or strongly agreed that they know where to find helpful health resources on the Internet, and over 90% reported having the skills needed to evaluate online health resources. Similarly, 88% reported being able to differentiate high- from low-quality online resources. Still, 12% of participants were undecided or disagreed that they could differentiate between high- and low-quality online health resources, and approximately 30% of participants were unsure or lacked confidence in using online information to make health-related decisions for self/family (see Figure 1).

### 3.3 Parent participants' technology use

Participants were active on the Internet, with the majority (*N* = 74, 63%) reporting 13 or more hours per week of Internet use (see Table 2). Most (87%, *N* = 100) participants reported spending one to four hours a week locating health information online, and 6% (*N* = 7) reported spending five or more hours per week seeking online health information. Yet 7% (*N* = 8) reported that they did not spend any time accessing online resources.

**Table 2:** Hours spent using the internet in a typical week (*N* = 118).

	Count (*N*=)	%
Hours spent per week using the Internet for general use		
0 h	1	1%
1–4 h	2	2%
5–8 h	16	14%
9–12 h	18	15%
13 + h	74	63%
Did not know	7	6%

Participants' online social media use (e.g., Facebook, Instagram, Snapchat, Twitter, LinkedIn) was predominantly a way to keep up to date (*N* = 91, 80%), communicate (*N* = 88, 77%), or to share their thoughts and images (*N* = 68, 60%) with family and friends (see Table 3).

**Table 3:** Activities that participants regularly conducted on social networking sites or apps (*N* = 113).

	Mean	*s*
Total number of activities participants regularly use social networking apps for	3.42	1.85
Activities conducted on social networking sites/apps	Count (*N*=)	%
Keep up to date with the activities of friends and family	91	80%
Communicate with friends and family	88	77%
Share or post your own thoughts, pictures or videos with friends and family	68	60%
Follow current events	66	58%
Learn about government programs or services (e.g., by following your municipality or an elected official on social networks)	52	46%
Share or post your own thoughts, pictures or videos publicly	18	16%
Other (e.g., work, follow parenting groups, follow musicians)	9	8%

Participants reported using smartphone devices (*N* = 108, 92.3%) and video calling apps (*N* = 84, 71.8%) as important tools in relation to their parenting role (see Table 4). When asked, 44% of participants (*N* = 67) reported the need to be skilled with computer technology to be successful at parenting. The belief that parenting success is reflected, in part, by technological skill was not related to participants' age (rpb = −0.09, *N* = 141, *p* = 0.28). Chi-square analyses indicated that there was no association between this finding and participants' education [*χ*2(4, *N* = 136) = 1.41, *p* = 0.84], annual income [*χ*2(4, *N* = 135) = 2.87, *p* = 0.58], employment status [*χ*2(4, *N* = 136) = 7.25, *p* = 0.12], or community size [*χ*2(3, *N* = 135) = 1.95, *p* = 0.58]. While participants who believed they needed to be skilled with technology to be successful at parenting reported a slightly higher average eHEALS score (*M* = 32.13, *s* = 4.37) compared to participants who did not share this belief (*M* = 31.82, *s* = 4.42), this difference was not statistically significant [*t*(107) = .35, *p* = .72].

**Table 4:** Types of technologies used by participants in relation to parenting (*N* = 120).

	Mean	Standard Deviation
Total number of different technologies used in relation to parenting	4.03	1.57
Technologies used in relation to parenting	Count (*N*=)	%
Smartphone (e.g., iPhone, Samsung Galaxy)	108	92.3%
Video calling (e.g., Skype, FaceTime, Google Hangouts)	84	71.8%
Laptop (e.g., MacBook)	81	69.2%
Tablet (e.g., iPad)	62	53.0%
TV (e.g., Apple TV)	57	48.7%
Cell phone	27	23.1%
Desktop	26	22.2%
eReader (e.g., Kindle, Kobo)	9	7.7%
Gaming system (e.g., Xbox, Play Station, Wii)	8	6.8%
Drone	5	4.3%
Other	3	2.6%
GoPro	2	1.7%

Participants were active online in relation to their parenting role. Almost 90% of participants reported using social media (e.g., Facebook, Twitter, Instagram), 85% used text messaging (e.g., iMessage, WhatsApp), and 77% accessed virtual communications (e.g., Skype, Facetime), respectively (see Table 5). Most participants (*N* = 88, 86.3%) reported four or fewer hours per day on social media for parenting purposes and almost 10% (*N* = 10) used social media up to eight hours per day. Participants were similarly active in their reported text messaging behaviors, with 85.3% (*N* = 88) reporting four or fewer hours per day of communication by texting, and about 6% reported up to 8 h per day. The use of online video and/or voice calls for four or fewer hours per day was a predominant communication strategy among participants (96.6%, *N* = 84). Given their significant use of social media and online communication, it was notable that only one participant cited concern about security or privacy issues.

**Table 5:** Number and type of parenting-related internet activities conducted by participants in a typical week (*N* = 116).

	Mean	Standard Deviation
Total number of Internet activities related to parenting participants conducted in a week	3.41	1.06
	Count (*N*=)	%
Activities related to parenting conducted on the Internet in a typical week		
Used social networking websites or apps	104	89.7%
Sent messages using an instant messaging app	98	84.5%
Made online voice calls or video calls	89	76.7%
Sent and received emails	81	69.8%
Uploaded self-created content on sharing websites (e.g., YouTube, Flickr) excluding social networking sites or apps	15	12.9%
Uploaded content to a blog or a personal website, excluding social networking websites or apps	9	7.8%

It makes sense that during the pandemic, when physical and social restrictions were heightened, participants relied on virtual strategies to manage and organize their daily parental activities. The types of online activities participants attributed to their parenting role included management of their financial affairs (*N* = 110, 96%), booking appointments (*N* = 86, 75%), and participating in training or learning opportunities (*N* = 58, 51%) (see Table 6). Participants also engaged in online shopping for purchasing parenting-related books/magazines (*N* = 31, 27.4%), toys/games (*N* = 76, 67.3%), food/groceries (*N* = 24, 21.2%), prescription drugs/health products (e.g., glasses) (*N* = 4, 3.5%), and non-prescription health products (*N* = 17, 15%).

**Table 6:** Number and type of online activities participants conducted in relation to parenting in previous three months (*N* = 114).

	Count (*N*=)	%
Types of online activities conducted in relation to parenting		
Conducted online banking	110	96%
Booked appointments	86	75%
Checked schedules or registered for classes (e.g., swimming lessons, fitness classes, movies)	71	62%
Taken informal training or learning (e.g., informal training tutorials, how-to videos, language learning apps)	58	51%
Searched for employment	27	24%
Taken formal training or learning through an organization or institution	25	22%

Participants also used connected (Internet of Things, IoT) home devices, with almost half (*N* = 47, 48%) reporting use of a video camera for security and/or infant monitoring. Over half of participants reported having a smart television (*N* = 72, 73.5%) or voice-activated systems (e.g., Google Home or Alexa) (*N* = 59, 60.2%) as part of their connected home system. Thirty-five percent (*N* = 40) of participants did not spend any time using connected (IoT) home devices in relation to parenting. Almost half of participants (*N* = 51, 44%) reported using connected home devices four or fewer hours per week, with 10% (*N* = 12) spending 13 or more hours per week using connected technology for parenting purposes, respectively.

Participants also expressed negative experiences related to their online (social media) engagement. The most prevalently reported negative experiences were loss of time (active online longer than anticipated), reduced physical activity, and lack of sleep. Participants also reported feeling anxious, depressed, and having difficulties concentrating related to their time online and especially social media activity (see Table 7). Over half (55%, *N* = 62) of participants took a break from using the Internet during the past 12 months because they felt they were using it too often or for too long.

**Table 7:** Number and type of negative experiences participants felt in past year as a result of social networking use (*N* = 115).

	Mean	*s*
Total number of negative experiences participants felt in the past year as a result of social networking use	3.19	2.24
	Count (*N*=)	%
Types of negative experiences felt as a result of social networking use		
Stayed online for longer periods than anticipated	91	79%
Lost sleep	49	43%
Felt anxious	49	43%
Had less physical activity	47	41%
Felt envious of the lives of others	37	32%
Felt frustrated or angry	30	26%
Had trouble concentrating on tasks or activities (e.g., school, work)	26	23%
Felt depressed	25	22%
Had relationship issues with friends or family	10	9%
Felt bullied or harassed	2	2%
I have not experienced any negative effects of social media	15	13%

## 4 Discussion

The COVID-19 context factored heavily into the experiences of pregnant and parenting families, and it is within these circumstances that we situate our findings on the intensification of parental digital technology use to seek out health information, connection, and support among our sample population (34, 35). We investigated parents' digital health literacy skill and their use of digital technologies using an online survey. There were no significant relationships between technology use, digital health literacy skill, and demographic variables. The majority of participants in this study represent a highly homogeneous group of parents who are female, highly educated, mostly employed, and with high income, creating limited statistical variance and narrowed representation of parents. Parenting before the pandemic was a gendered activity, with women performing a disproportionately larger share of parental tasks than men, and this pattern continued into the pandemic (36) and is echoed in the gendered nature of participants in this current study. The choice of an online survey reflects consideration of efficiency of time, resources, data quality, and respect of the existing public health restrictions of the time (37). However, in this study, the homogenous nature of participants reflected a narrowed perspective of white, educated, and seemingly advantaged women's online behaviours. PEW researchers determined that individuals who are older, racialized, and with less than high school education are not well-represented within online survey data (37, 38). They caution researchers about the use of online-only data collection tools and the potential of associated errors and biases. We highlight this as an opportunity for researchers to consider the use of multiple data collection strategies to enhance the diversity of potential participants.

Research from pre-COVID-19 demonstrated persistently high use of digital technologies during the transition to parenting (2); therefore, the ubiquitous use among a highly educated sample during a time of decreased access to face-to-face primary health care service may not in itself be surprising. During COVID-19, when essential health and social services were rapidly moved into digital spaces, sometimes exclusively, it became normative (if not encouraged) for individuals to seek online for health resources, social connection, and other needed services. The impetus to acquire health information was not confined to discrete appointments between participants and their health care providers, but spanned time and place. Participants in this study predominantly used their smartphones to connect with family and friends, for online training, to access information, to book appointments, to shop via social media, for texting, and for online video and/or voice calls.

The obligation to be online was propelled by the COVID-19 pandemic, and among the sample in this study, participants reported having the digital health capacity and personal resources (e.g., infrastructure) to adjust to the demands of the pandemic and to move to online provision of health services and supports. Among participants, 44% reported the need to be skilled with computers and new technology to be successful at parenting. Additionally, participants claimed higher than the Canadian average self-appraisal skills for online information seeking, as over 90% reported sufficient digital health literacy skills to discern credible from non-credible online information, and less than 10% of participants self-reported inadequate or problematic digital health literacy skills. The mean digital health literacy score for participants in this study was above the Canadian national average score of 27.55 during the COVID-19 pandemic (14). When compared to the reported digital health literacy skill of individuals of the same age, participants in the current study reported higher digital health literacy skill than other Canadians aged 16–34 years in the national survey (13). Other research has demonstrated that parents of adolescents with type 1 diabetes also reported higher than the national average scores for health literacy (39). Researchers hypothesized that parents of children with diabetes had been dealing with their child's chronic disease for several years, which reflected in their greater health literacy skill. This may be a similar consideration for the sample of parents in the current study, where new parents, especially mothers, take on the role of information curation related to prenatal and early postnatal care (2).

Yet despite the majority of participants claiming they had the skills necessary to locate resources online and to determine the quality of the information they encounter, 30% of participants self-reported a lack of confidence in their abilities to make health-related decisions based on the information they retrieved. This finding is consistent with previous survey research (*N* = 402) conducted by Paige et.al. (2017) wherein 75% of participants reported having the skills to evaluate online health resources; however, when it came to confidence to evaluate the quality of and make decisions to act on this information, this number dropped to 60% (40). There are two important opportunities here: (1) to position online health information seeking and digital health literacy as critical topics for basic health education, and (2) for health care providers to adjust their workflow to include inquiry into parents’ online health information seeking to elevate shared decision-making practices.

Findings from the current study suggest that health care provider inquiry into parents' online activity is relevant to their physical and mental health. Participants associated their online activity with a number of negative health consequences: 40% of participants reported lack of sleep, physical activity, and anxiety, and over 20% reported feelings of depression related to their online activity. Meaney et al. (2022) found among pregnant women residing in the United States low levels of social support, restrictions on maternity services, and limited face-to-face interactions with health care professionals during the pandemic, along with elevated mental health concerns and stress among this population (34). These findings also align with results from an online survey conducted by Davenport et al. (2020) who reported a rapid acceleration of signs of clinical depression among pregnant and postpartum women during the COVID-19 pandemic once isolation policies were in place (35). The number of parents who sustained their online connection regardless of the potential for negative impacts may be accounted for by a need to reduce their social isolation and to feel a part of the social world outside the boundaries of their residence. This may be particularly true at the intersection of the postpartum period, a time already marked by increased isolation and lower mental health status, and a global pandemic wherein sweeping changes were felt in each domain of the transition to parenting (34, 41). These findings are consistent with participants in the current study; many reported curbing their online social media use to reduce the reported negative outcomes, while 45% retained their intensity of use regardless of the negative outcomes.

Given their substantial use of social media and online communication, it is notable that only one participant cited concern about security or privacy issues. A discussion around privacy and new imaginings of “privacy” may be warranted; it requires rethinking about how privacy is negotiated and how the concept of privacy is currently contextualized. Research conducted with older adolescents and young adults found that they expressed little concern about the collection and future use of their personal data while also showing limited knowledge of the business practices involved in using such information for commercial purposes (42, 43).

With the assumption that face-to-face maternity services will not return to in-person pre-COVID-19 levels, we must consider alternative and multiple strategies to connect with a diversity of parents to understand their needs and to provide care. Important considerations for clinicians include integrating discussions within client-provider encounters to collaboratively consider strategies to mitigate the potential for negative health consequences from online activities related to parenting. Findings draw attention to the importance of an awareness of digital health literacy skills within health professions' education. Opportunities within the clinical encounter should be identified to provide curated online health information resources, recognizing the likelihood of their clients seeking health information from online sources. Consideration should be given to telehealth consultation in support of prenatal and postpartum care, lactation practices (e.g., telelactation), and tele-mental health services that may ease access care for parents dealing with postpartum depression. Furthermore, there is an important role within Public Health services to support and educate parents about effective online information seeking and the important influence digital health information seeking has on individuals during the transition to parenting. At minimum, public health pre-natal class instruction should provide insight into effective strategies for online information seeking and quality checklists to support parents' evaluation of the many online health related apps and products marketed to parents. Significant considerations for ongoing health care services include the need for structural changes to ensure health inequities are not exacerbated among populations who lack the resources (e.g., data plan, smartphone/devices, literacy skills) to harness digital technologies in ways that promote health.

### 4.1 Future directions

Based on findings from this current study, further research is needed to examine whether there are greater or different information needs related to different time periods within pregnancy and early parenting. For instance, health information needs may increase for a pregnant person who is post-dates or who is in the very early stages of new parenting. There may be different information needs among individuals depending on their primary health care provider (e.g., midwife versus obstetrician) and dependent upon the relationship of trust between them. Exploring the longitudinal impacts of digital health literacy skills on parenting and health outcomes would inform clinical and public health best practices.

While the Internet constitutes a vast resource of information, the initial promise of the Internet as a “level playing field” where everyone would have equal opportunity for access has not been fully realized (44, 45). Research is needed to critically look at the benefits and challenges to online and digital health resources to support equitable access to health information services and supports. Many Canadians, particularly marginalized populations and isolated communities, remain at a disadvantage in terms of access to digital spaces (44, 46). In light of findings reported from a study on the impact of COVID-19 on experiences of care among pregnant women, telehealth was noted by women to confer some benefits; for example, eliminating travel time to health care providers for antenatal or postnatal appointments. However, women expressed concern that critical information would be undetected during telehealth appointments, such as pregnancy or postpartum complications (47).

Research opportunities also exist to explore the digital parenting practices among a diverse representation of family structures, fathering, newcomers, English as an additional language, families living in rural and remote communities, and among populations with documented lower health literacy and digital health literacy. Additionally, research is needed to investigate the impact of digital health literacy skills on parents' decision-making practices and health outcomes.

### 4.2 Limitations

This study comprised a non-experimental, cross-sectional design using a non-probability, convenience sampling strategy; therefore, there are limitations to generalizability based on the sample. The homogeneity of the participant sample produced a sampling bias. This resulted in participants who were primarily white, cisgender, and who lived within heteronormative family structures limits the applicability of results outside these demographic characteristics. There also appeared to be reticence from fathers to participate in this study, which limited our understanding of the use of digital technologies during the transition to parenting to the maternal perspective. Future studies that are created with input and in partnership with fathering and LGBT + social communities may support recruitment of these groups of parents. As recruitment and data collection were exclusively online given the COVID-19 context, using an online survey may have limited opportunities to reach potential participants not as proficient in digital technologies or digital spaces.

## 5 Conclusion

The COVID-19 pandemic has reinforced the need for and importance of effective and equitable digital health services. Parents in this study are characteristic of white, well-educated mothers with adequate income and moderate to high digital health literacy proficiency to support their participation in online digital health settings. The importance and “taken for granted” use of mobile devices to support parenting within the digital health setting was motivated by the COVID-19 context but also reflects the intensity of information needs among new parents. Despite the normative online activity among parents, many experienced negative consequences, resulting in digital vacations for some. Important opportunities exist within clinical practice and among parenting groups to proactively address the physical and mental health implications of digital parenting practices. Equally important are the opportunities to insert into the clinical workflow inquiries into parents' online information-seeking behaviours, and to include digital health literacy as part of prenatal/postnatal health education initiatives.

## Data Availability

The raw data supporting the conclusions of this article will be made available by the authors, without undue reservation.
